# *BBOX1‐AS1* contributes to colorectal cancer progression by sponging hsa‐*miR‐361‐3p* and targeting *SH2B1*

**DOI:** 10.1002/2211-5463.12802

**Published:** 2022-03-26

**Authors:** Jiasheng Liu, Jie Zhu, Zhe Xiao, Xufeng Wang, Jianfei Luo

**Affiliations:** ^1^ Department of Gastrointestinal Surgery Renmin Hospital of Wuhan University China; ^2^ Information Section Armed Police Hubei Provincial Corps Hospital Wuhan China

**Keywords:** *BBOX1‐AS1*, colorectal cancer, *miR‐361‐3p*, *SH2B1*

## Abstract

Colorectal cancer (CRC) is the third main cause of cancer‐relevant deaths worldwide, and its incidence has increased in recent decades. Previous studies have indicated that certain long noncoding RNAs (lncRNAs) have regulatory roles in tumor occurrence and progression. Often, lncRNAs are competitive endogenous RNAs that sponge microRNAs to up‐regulate mRNAs. Here, we examined the role of a novel lncRNA gamma‐butyrobetaine hydroxylase 1 antisense RNA 1 (*BBOX1‐AS1*) in CRC. We observed that *BBOX1‐AS1* is overexpressed in CRC cell lines, and *BBOX1‐AS1* knockdown enhances cell proliferation, migration and invasion while reducing cell apoptosis. *miR‐361‐3p* is present at a low level in CRC and is negatively modified by *BBOX1‐AS1*. Moreover, *miR‐361‐3p* was validated to be targeted by *BBOX1‐AS1*. Src homology 2 B adaptor protein 1 (*SH2B1*) was notably upregulated in CRC cell lines and was identified as a downstream gene of *miR‐361‐3p*. In addition, we found that *miR‐361‐3p* amplification can suppress the expression of *SH2B1*. Finally, data from rescue assays suggested that overexpression of *SH2B1* counteracted *BBOX1‐AS1* silencing‐mediated inhibition of CRC progression. In conclusion, *BBOX1‐AS1* promotes CRC progression by sponging hsa‐*miR‐361‐3p* and up‐regulating *SH2B1*.

## Graphical abstract

Here, we provide evidence to suggest that the *BBOX1‐AS1*/*miR‐361‐3p*/*SH2B1* axis promotes colorectal cancer cell proliferation, migration, and invasion, and inhibits cell apoptosis.

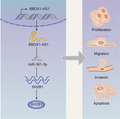

**Abbreviations**
*BBOX1‐AS1*
: gamma‐butyrobetaine hydroxylase 1 antisense RNA 1
CCK‐8: Cell Counting Kit‐8
ceRNA: competing endogenous RNA
CRC: colorectal cancer
EMT: epithelial–mesenchymal transition
GAPDH: glyceraldehyde‐3 phosphate dehydrogenase
lncRNA: long noncoding RNA
miRNA: microRNA
Mut: mutant
NC: negative control
RIP: RNA immunoprecipitation
SD: standard deviation
sh‐*BBOX1‐AS1*
: short hairpin RNA targeting *BBOX1‐AS1*
*SH2B1*
: Src homology 2 B adaptor protein 1
WT: wild‐type

Colorectal cancer (CRC) is known as the third main cause of cancer‐relevant deaths worldwide, and its occurrence continues to increase in recent decades [1]. Although great progress has been made in medical strategy, the prognosis of patients with CRC is still largely disappointing on account of the distant metastasis of tumor in advanced stage [2]. Consequently, it is greatly urgent to search the molecular mechanisms underlying CRC progression and to explore potential therapies for patients with CRC.

Long noncoding RNAs (lncRNAs) are a group of RNAs whose length is >200 nucleotides and limited to code proteins [3]. They are reported to exert key roles in the regulation of genetic transcription and pathogenesis of various tumors [4, 5]. For example, lncRNA HOXD‐AS1 was confirmed to drive the metastasis of melanoma cells by restraining the expression of RUNX3 [6]. In addition, high expression of HULC resulted in a poor prognosis and facilitated the prostate cancer progression via regulating the epithelial–mesenchymal transition (EMT) process [7]. It was widely recognized that lncRNA could interact with microRNAs (miRNAs) via functioning as competing endogenous RNA (ceRNA) that secluded miRNAs leading to suppression of their modulatory role in target mRNAs [8]. Several reports have shown that lncRNAs, as ceRNA, impacted tumor initiation and progression, reflecting a new regulatory mechanism at posttranscriptional level. For instance, lncRNA TUG1 was identified as an oncogene in osteosarcoma and enhanced osteosarcoma cell growth through modulating the expressions of miR‐212‐3p and FOXA1 [9]. lncRNA PVT1‐5 was discovered to participate in the regulation of lung cancer cell proliferation by sponging miR‐126 and targeting SLC7A5 [10]. In addition, numerous lncRNAs are validated to serve as a ceRNA in CRC progression. SOX21‐AS1 functioned as a sponge for miR‐145 to boost CRC tumorigenesis through targeting MYO6 [11]. HIF1A‐AS2 exerted a promotive effect on CRC progression and EMT formation by regulating the miR‐129‐5p/DNMT3A axis [12]. Up‐regulated in colorectal cancer liver metastasis (UICLM) enhanced liver metastasis of CRC via functioning as a ceRNA for miRNA‐215 to modify ZEB2 expression [13]. gamma‐Butyrobetaine hydroxylase 1 antisense RNA 1 (*BBOX1‐AS1*), a novel lncRNA, has not been investigated in cancers.

In this study, we devoted to probing the biological role and mechanism of *BBOX1‐AS1* in CRC. It was found that *BBOX1‐AS1* drives CRC progression by sponging hsa‐*miR‐361‐3p* and up‐regulating Src homology 2 B adaptor protein 1 (*SH2B1*). This discovery provides a promising theoretical basis for the exploration of CRC therapeutic strategies.

## Materials and methods

### Cell culture

CRC cell lines (GEO, SW480, HCT116 and LOVO) and the human colorectal mucosal cell line (FHC) were gained from Chinese Academy of Sciences (Beijing, China). A Dulbecco’s modified Eagle’s medium (Corning Life Sciences, Tewksbury, MA, USA) mixed with 10% FBS (Warbison Technology, Beijing, China), 100 mg·mL^−1^ penicillin and streptomycin (Invitrogen, Karlsruhe, Germany) were used for culturing the earlier cells in a humid atmosphere at 37 °C with a 5% CO_2_.

### Cell transfection

Cells were plated into six‐well plates under a density of 70–80%. The short hairpin RNAs targeting *BBOX1‐AS1* (sh‐*BBOX1‐AS1*#1/2) or their negative control (sh‐NC) purchased from Genechem (Shanghai, China) were transfected into GEO and HCT116 cells. The overexpression plasmid pcDNA3.1/*SH2B1* and the empty pcDNA3.1 vector were obtained from Genechem. The *miR‐361‐3p* mimics or NC mimics were constructed by ZonHon Biopharma Institute (Changzhou, Jiangsu, China). Lipofectamine 2000 (Invitrogen) was performed for transfection that was conducted 48 h later.

### Quantitative real‐time PCR

Total RNA was separated from cultured cells by TRIzol reagent (Thermo Fisher Scientific, Waltham, MA, USA) based on the manufacturer’s guides. Reverse Transcription Kit (Takara, Tokyo, Japan) was applied to reverse transcription. The quantitative real‐time PCR analysis was conducted by SYBR Green Premix PCR Master Mix (Roche, Mannheim, Germany) via ABI HT9600 (Applied Biosystems, Foster City, CA, USA). The relative RNA level was detected using the $2^{-\Delta \Delta C_{t}}$ method. In addition, glyceraldehyde‐3 phosphate dehydrogenase (GAPDH) or U6 served as internal control.

### Western blot

Total protein was extracted from lysates added with protein inhibitors. Extracted proteins were isolated by SDS/PAGE (Boster Biological Technology, Los Angeles, CA, USA) and then transferred into poly(vinylidene fluoride) (East Fluorine Chemical Technology, Shanghai, China). After being blocked with skim milk, the membranes were incubated with primary antibodies at 4 °C overnight. The primary antibodies for our experiment were *SH2B1* Ig (ab228828; Abcam, Cambridge, UK) and GAPDH Ig (ab8245; Abcam). GAPDH was seen as a reference. The protein blot was visualized by enhanced chemiluminescence system (GE Healthcare, Chicago, IL, USA).

### Colony formation assay

At first, about 800 cells were seeded into six‐well plates after transfection. After being incubated in a chamber under an environment of 5% CO_2_ at 37 °C for 2 weeks, paraformaldehyde (Solarbio, Beijing, China) was used to fix the colonies for 10 min and dyed with crystal violet (Beyotime, Nantong, Jiangsu, China) for 5 min. Colonies were counted manually.

### Luciferase reporter assay

The wild‐type (WT) binding sites of *SH2B1*/*BBOX1‐AS1* and the mutant (Mut) binding sites of *SH2B1*/*BBOX1‐AS1* with *miR‐361‐3p* were subcloned into pmirGLO dual‐luciferase vector to construct *SH2B1*‐WT/*BBOX1‐AS1*‐WT and *SH2B1*‐Mut/*BBOX1‐AS1*‐Mut plasmids. Then *miR‐361‐3p* mimics or NC mimics were cotransfected into GEO or HCT116 cells, respectively. After the transfection of 48 h, luciferase activity was detected.

### Transwell invasion assay

The aim of transwell assay was to test the invasion ability of GEO and HCT116 cells. Cells were seeded on the upper chamber, which the membrane precoated with Matrigel matrix gel (BD Biosciences, San Jose, CA, USA). The upper chamber was applied with serum‐free medium, and the lower chamber was added with 10% FBS. Invaded cells were fixed with methanol (Ceran Technology, Chengdu, China) and dyed with crystal violet after the scheduled time. Five fields at random were counted, and invaded cells were observed via a microscope.

### Cell Counting Kit‐8 assay

The GEO or HCT116 cells (1 × 10^3^ cells/well) were seeded in six‐well plates in a complete medium and cultured for 0, 24, 48, 72 and 96 h. Then Cell Counting Kit‐8 (CCK‐8; Dojindo Molecular Technologies, Tokyo, Japan) solution was added and incubated for an additional 2 h. Relative cell viability was determined using an EL × 800 micro‐immuno analyzer (Bio‐Tek, Winooski, VT, USA).

### TUNEL staining assay

TUNEL assay was conducted to analyze the level of cell apoptosis in GEO and HCT116 cells. After TUNEL staining, the GEO or HCT116 cells were stained using 4′,6‐diamidino‐2‐phenylindole (Sigma‐Aldrich, Downtowner, St. Louis, MO, USA). After that, relative fluorescence intensity was observed using a laser scanning confocal microscope (Olympus, Tokyo, Japan).

### Flow cytometry analysis

Annexin V‐FITC/PI Apoptosis kit (Invitrogen) was used to measure the apoptosis of GEO and HCT116 cells. In brief, the apoptotic cells were cleaned using PBS (Solarbio) and resuspended. Later, 70% ethanol cooled by ice was used to fix cells. At last, the apoptosis rate was evaluated by FACSCalibur flow cytometer (BD Biosciences).

### Cell scratch test

GEO or HCT116 cells were centrifuged, and the cell suspension was cultured in six‐well plates for 24 h. After the degree of cell fusion reached 80–90%, the transfer gun was used to draw some scratches on the back of each plate with the same force. Then the plates were washed thrice with PBS (Thermo Fisher Scientific). Images were photographed at 0 and 24 h on motic images advanced 3.2 software (Motic Asia, Hong Kong, China).

### Subcellular fractionation assay

In line with the manufacturer’s protocol, PARIS Kit (Invitrogen) was used to isolate nuclear and cytoplasmic fractions. Relative *BBOX1‐AS1*, GAPDH (cytoplasmic control) and U6 (nuclear control) expressions in the cytoplasm or nuclear of GEO and HCT116 cells were assessed by quantitative real‐time PCR.

### RNA immunoprecipitation assay

Imprint RNA immunoprecipitation (RIP) kit (Millipore, Bedford, MA, USA) following the specification was used for RIP assays. In brief, GEO or HCT116 cells lysed in RIP lysis buffer were incubated with anti‐Ago2 antibody or anti‐IgG antibody that was preattached to magnetic beads. The quantitative real‐time PCR was applied for the detection of immunoprecipitated RNA.

### RNA pull‐down assay

RNA pull‐down assay was chosen to confirm the specific binding of *BBOX1‐AS1* and hsa‐*miR‐361‐3p*. The acquired cell lysates were treated with Bio‐*miR‐361‐3p*‐WT/Mut or Bio‐miR‐NC, together with streptavidin‐labeled magnetic beads. The compound was analyzed by the quantitative real‐time PCR.

### Statistical analysis

graphpad prism 7.0 software (La Jolla, CA, USA) was applied for statistical analysis, and the experimental data from at least three independent experiments were shown as mean values ± standard deviation (SD). The Student’s *t*‐test or one‐way/two‐way ANOVA was used for analyzing the comparisons of groups. Statistical significance was obtained when *P* < 0.05.

## Results

### *BBOX1‐AS1* is overexpressed in CRC cell lines and facilitates CRC cell proliferation, migration and invasion while inhibiting apoptosis

**Fig. 1:**
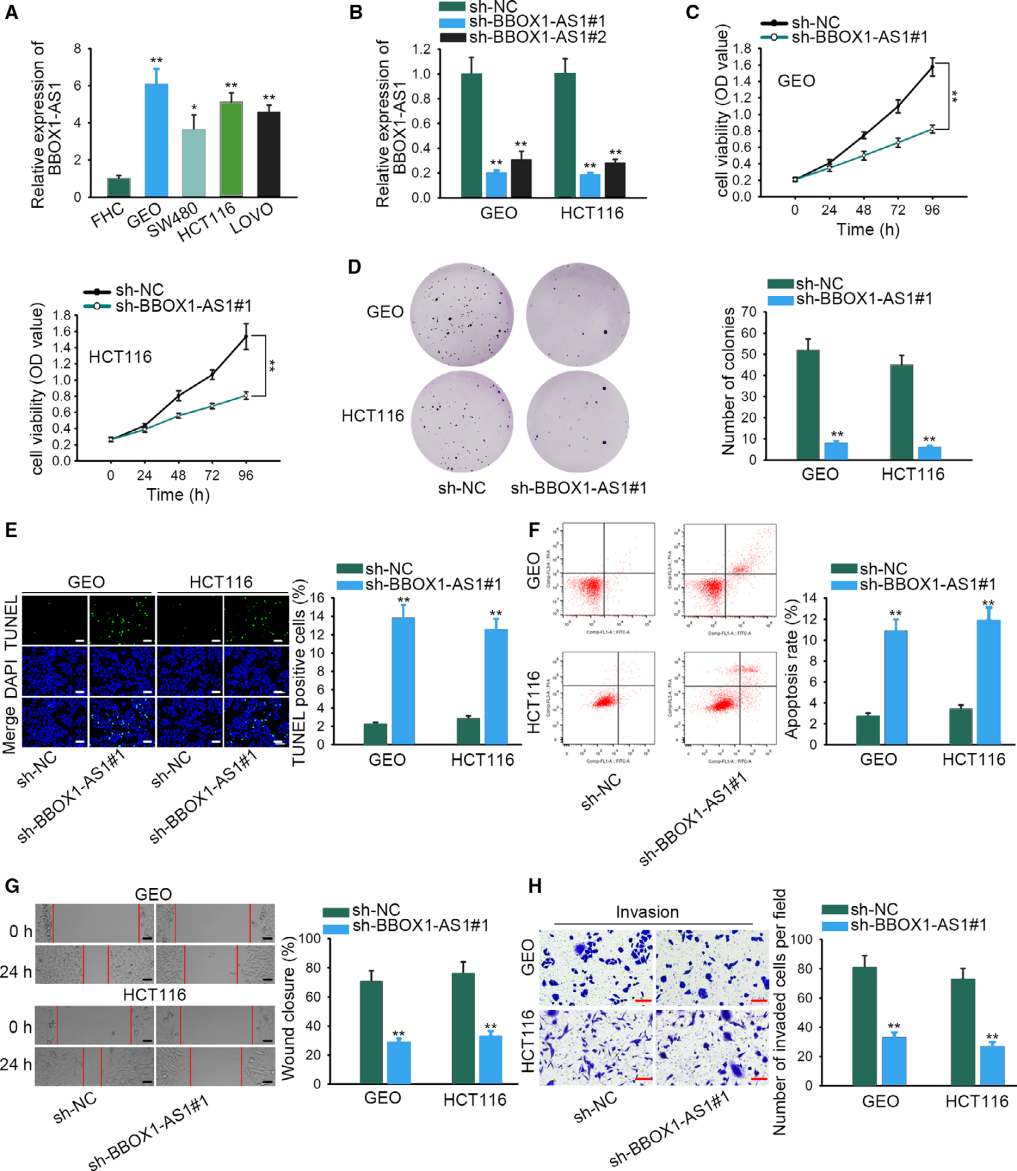
*BBOX1‐AS1* is up‐regulated in CRC cell lines and promotes CRC cell proliferation, migration and invasion while inhibiting apoptosis. (A) *BBOX1‐AS1* expression in CRC cell lines (GEO, SW480, HCT116, and LOVO) and colorectal mucosal cell line (FHC) was detected (*n* = 4). One‐way ANOVA. (B) The transfection efficiency of sh‐*BBOX1‐AS1* was tested (*n* = 4). One‐way ANOVA. (C, D) CCK‐8 (two‐way ANOVA) and colony formation assays (Student's *t*‐test) showed the proliferation in GEO and HCT116 cells, which were transfected with sh‐*BBOX1‐AS1* and sh‐NC (*n* = 4). (E, F) TUNEL assay (scale bars, 150 μm) and flow cytometry analysis were performed to assess the apoptosis of GEO and HCT116 cells after *BBOX1‐AS1* was knocked down (*n* = 4). Student’s *t*‐test. (G) With the transfection of sh‐*BBOX1‐AS1*, representative images of migrated cells were illustrated through wound healing assay (scale bars, 160 μm; *n* = 4). Student’s *t*‐test. (H) Transwell assay (scale bars, 180 μm) was applied to evaluate cell invasion in GEO and HCT116 cells, which depleted *BBOX1‐AS1* (*n* = 4). Student’s *t*‐test. The error bars indicate SD. *P < 0.05, **P < 0.01 suggested a statistically significant difference in comparison with the control group.

First, quantitative real‐time PCR analysis was used for detecting *BBOX1‐AS1* expression in CRC cell lines (GEO, SW480, HCT116, LOVO), and one colorectal mucosal cell line (FHC) was used as a reference. As a result, a high level of *BBOX1‐AS1* was found in CRC cell lines (Fig. 1A, *P* < 0.05, *P* < 0.01). To investigate the function of *BBOX1‐AS1* on CRC progression, we transfected GEO and HCT116 cells with sh‐*BBOX1‐AS1* for knocking down *BBOX1‐AS1* expression (Fig. 1B, *P* < 0.01), and sh‐*BBOX1‐AS1*#1 was chosen for the subsequent assays because of the better interfering efficiency. The results from CCK‐8 and colony formation assays disclosed that silenced *BBOX1‐AS1* significantly restrained the proliferation in GEO and HCT116 cells (Fig. 1C,D, *P* < 0.01). The apoptosis in GEO and HCT116 cells was evidently hastened with the transfection of sh‐*BBOX1‐AS1* through TUNEL assay and flow cytometry analysis (Fig. 1E,F, *P* < 0.01). Later, wound healing assay delineated that *BBOX1‐AS1* deficiency dramatically suppressed the migration in GEO and HCT116 cells (Fig. 1G, *P* < 0.01). Lastly, transwell assay was conducted to assess cell invasion. As expected, sh‐*BBOX1‐AS1* transfection effectively inhibited the invasion in GEO and HCT116 cells (Fig. 1H, *P* < 0.01). Taken together, *BBOX1‐AS1* was overexpressed in CRC cell lines and facilitated CRC cell proliferation, migration and invasion while inhibiting apoptosis.

### *BBOX1‐AS1* acts as a sponge for *miR‐361‐3p*

**Fig. 2:**
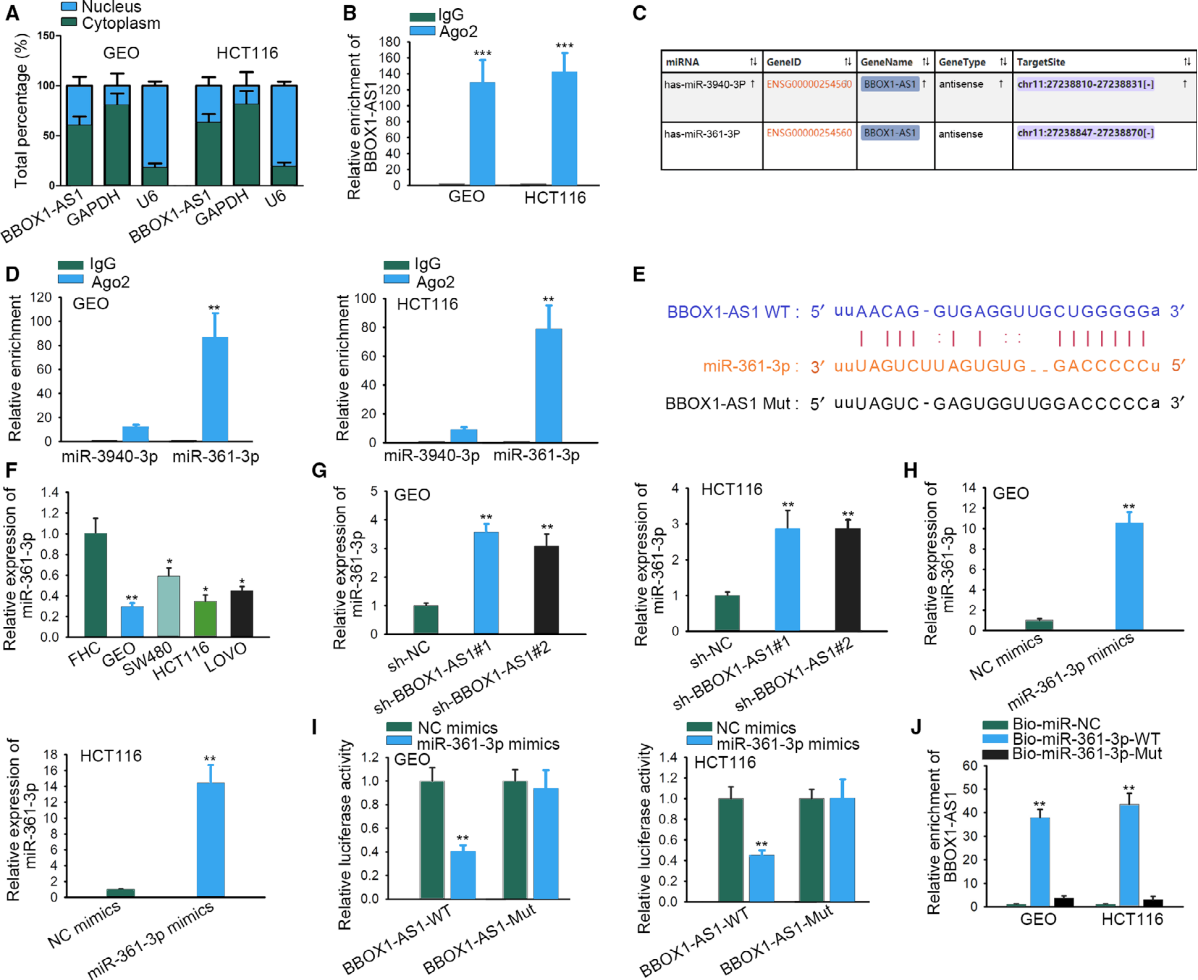
*BBOX1‐AS1* acts as a sponge for *miR‐361‐3p*. (A) Subcellular fractionation assay was used to determine the distribution of *BBOX1‐AS1* in GEO and HCT116 cells (*n* = 4). (B) RIP assay confirmed the combination between *BBOX1‐AS1* and RNA‐induced silencing complex (*n* = 4). Student’s *t*‐test. (C) The potential miRNAs predicted from starbase for *BBOX1‐AS1* were displayed. (D) The enrichment of potential miRNAs in the beads conjugated with the antibody of Ago2 or IgG was measured by RIP assay (*n* = 4). Student’s *t*‐test. (E) The binding site between *miR‐361‐3p* and *BBOX1‐AS1* was presented. (F) Quantitative real‐time PCR analysis was used to test *miR‐361‐3p* expression in CRC cell lines and the colorectal mucosal cell line (*n* = 4). One‐way ANOVA. (G) The expression of *miR‐361‐3p* in *BBOX1‐AS1*‐silenced cells was detected (*n* = 4). One‐way ANOVA. (H) The expression of *miR‐361‐3p* in CRC cells transfected with *miR‐361‐3p* mimics was detected (*n* = 4). Student’s *t*‐test. (I) Luciferase reporter assay was conducted to confirm the interaction between *BBOX1‐AS1* and *miR‐361‐3p* (*n* = 4). Student’s *t*‐test. (J) *BBOX1‐AS1* was verified to combine with *miR‐361‐3p* by RNA pull‐down assay (*n* = 4). One‐way ANOVA. The error bars indicate SD. *P < 0.05, **P < 0.01, ***P < 0.001 revealed a statistically significant difference in comparison with the control group.

It was reported that lncRNAs sponged miRNAs and thereby regulated mRNAs expression to mediate biological processes at posttranscriptional level. Using subcellular fractionation assay, we found the main expression of *BBOX1‐AS1* in the cytoplasm of GEO and HCT116 cells (Fig. 2A), indicating posttranscriptional regulation of *BBOX1‐AS1* in CRC. RIP assay indicated that *BBOX1‐AS1* was specifically enriched in Ago2 immunoprecipitate (Fig. 2B, *P* < 0.001). These results provided a hypothesis that *BBOX1‐AS1* might be a ceRNA in CRC. As illustrated in Fig. 2C, two potential miRNAs (miR‐3940‐3p, *miR‐361‐3p*) were predicted to be sponged by *BBOX1‐AS1* (Fig. 2C). Then RIP assay displayed that the enrichment of *miR‐361‐3p* in beads conjugated with Ago2 antibodies was much higher (*P* < 0.01), whereas that of miR‐3940‐3p was not statistically significant (Fig. 2D). Hence we predicted that *miR‐361‐3p* acted as a target of *BBOX1‐AS1*. With the use of starbase (http://starbase.sysu.edu.cn/), the binding site between *BBOX1‐AS1* and *miR‐361‐3p* was presented in Fig. 2E. Subsequently, it was found that *miR‐361‐3p* was down‐regulated in CRC cell lines (Fig. 2F, *P* < 0.05, *P* < 0.01). In addition, the silence of *BBOX1‐AS1* could increase *miR‐361‐3p* expression (Fig. 2G, *P* < 0.01). Besides, transfection efficiency of *miR‐361‐3p* mimics was detected, and results showed that *miR‐361‐3p* expression was prominently elevated with the transfection of *miR‐361‐3p* mimics (Fig. 2H, *P* < 0.01). Luciferase reporter assay delineated that relative luciferase intensity of WT *BBOX1‐AS1* was significantly reduced with the treatment of *miR‐361‐3p* mimics (*P* < 0.01), whereas no remarkable difference was shown in that of Mut type (Fig. 2I). In addition, RNA pull‐down assay with biotinylated *miR‐361‐3p* implied that *BBOX1‐AS1* was pulled down by Bio‐*miR‐361‐3p*‐WT (Fig. 2J, *P* < 0.01). All data expounded that *BBOX1‐AS1* acted as a sponge for *miR‐361‐3p*.

### *SH2B1* is a target gene of *miR‐361‐3p*

**Fig. 3:**
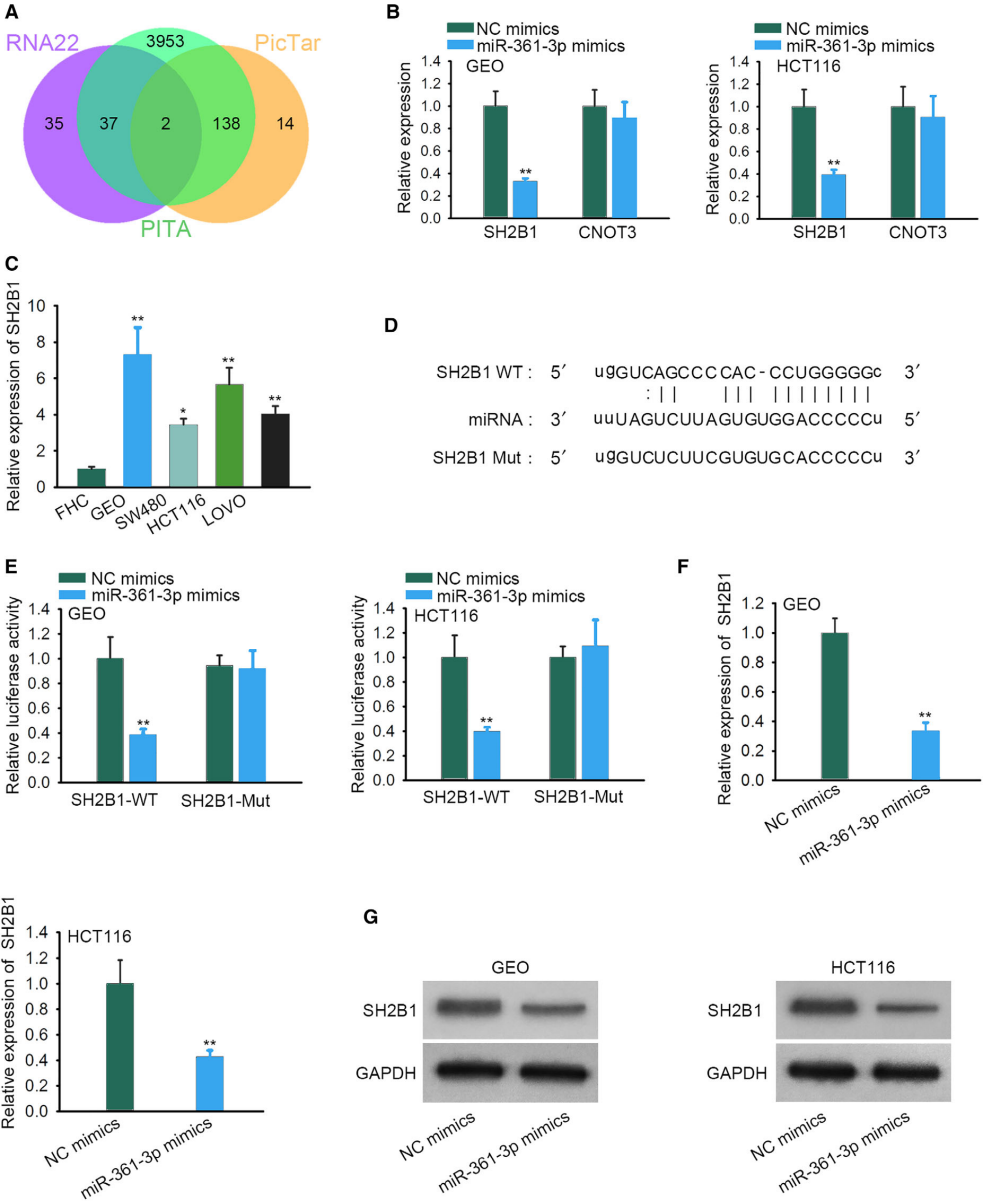
*SH2B1* is a target gene of *miR‐361‐3p*. (A) The potential target genes of *miR‐361‐3p* were predicted from three databases (PITA, RNA22, and PicTar). (B) Quantitative real‐time PCR analysis was used to test the expressions of potential target genes in CRC cells transfected with *miR‐361‐3p* mimics (*n* = 4). Student’s *t*‐test. (C) *SH2B1* expression level was measured in CRC cell lines and the colorectal mucosal cell line (*n* = 4). One‐way ANOVA. (D) The predicated binding sites of *miR‐361‐3p* on *SH2B1* 3′ UTR were demonstrated. (E) Luciferase reporter assay was performed to verify the interaction between *miR‐361‐3p* and *SH2B1* (*n* = 4). Student’s *t*‐test. (F, G) The effect of *miR‐361‐3p* mimics on *SH2B1* mRNA and protein levels was estimated (*n* = 4). Student’s *t*‐test. The error bars indicate SD. *P < 0.05, **P < 0.01 implied a statistically significant difference in comparison with the control group.

To support the hypothesis of a ceRNA mechanism, we then explored the target gene of *miR‐361‐3p*. It was predicted from three databases (PITA, RNA22, and PicTar) that *SH2B1* and CNOT3 were the potential downstream molecules for *miR‐361‐3p* (Fig. 3A). Quantitative real‐time PCR analysis depicted the inhibitory effect of *miR‐361‐3p* elevation on *SH2B1* (*P* < 0.01), rather than on CNOT3 (Fig. 3B). Therefore, *SH2B1* was chosen for the subsequent experiments. Importantly, high *SH2B1* level was detected in CRC cell lines (Fig. 3C, *P* < 0.05, *P* < 0.01). Moreover, *SH2B1* 3′ UTR was predicted to possess a binding site matched with the sequence of *miR‐361‐3p* (Fig. 3D). Luciferase reporter assay later confirmed that *miR‐361‐3p* up‐regulation overtly lessened the luciferase activity of *SH2B1*‐WT (*P* < 0.01) but had no effect on that of *SH2B1*‐Mut (Fig. 3E). Finally, we found that *miR‐361‐3p* overexpression markedly reduced *SH2B1* mRNA expression (*P* < 0.01) and protein level (Fig. 3F,G). Hence *SH2B1* was validated as a downstream gene of *miR‐361‐3p*.

### *BBOX1‐AS1* promotes CRC progression by sponging *miR‐361‐3p* and up‐regulating *SH2B1*

**Fig. 4:**
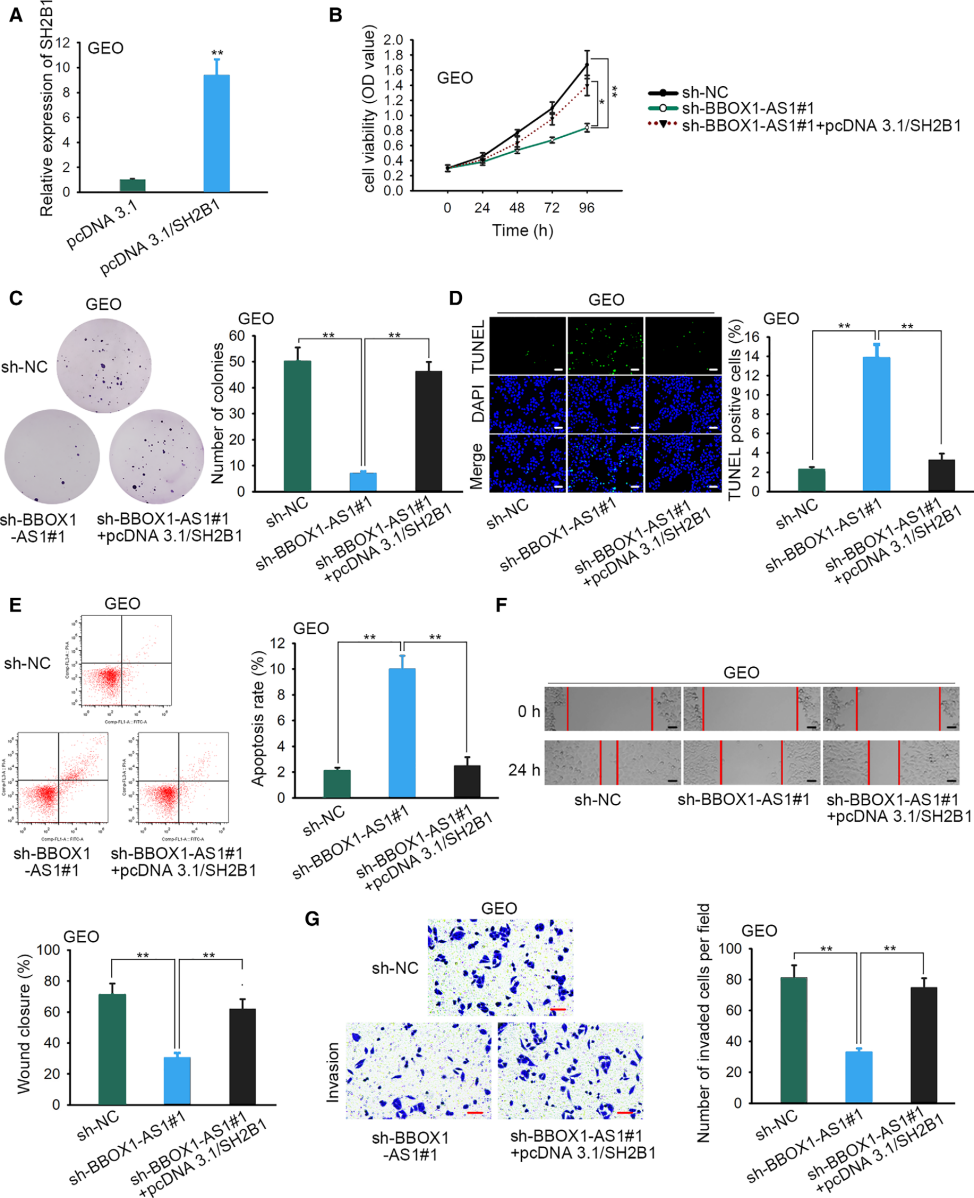
*BBOX1‐AS1* promotes CRC progression by sponging *miR‐361‐3p* and up‐regulating *SH2B1*. (A) Transfection efficiency of pcDNA3.1/*SH2B1* was detected (*n* = 4). Student’s *t*‐test. (B, C) The proliferation in CRC cells was determined with the transfection of indicated plasmids through CCK‐8 (two‐way ANOVA) and colony formation assays (one‐way ANOVA) (*n* = 4). (D, E) TUNEL assay (scale bars, 150 μm) and flow cytometry analysis were applied to evaluate cell apoptosis in each group (*n* = 4). One‐way ANOVA. (F) Wound healing assay (scale bars, 160 μm) was used to confirm the migration of cells transfected with appointed plasmids (*n* = 4). One‐way ANOVA. (G) Invasive cells in each group were shown by transwell assay (scale bars, 180 μm) (*n* = 4). One‐way ANOVA. The error bars indicate SD. *P < 0.05, **P < 0.01 disclosed a statistically significant difference in comparison with the control group.

In line with the earlier results, it was speculated that *BBOX1‐AS1* might serve as a ceRNA and regulate the *miR‐361‐3p*/*SH2B1* axis in the progression of CRC. To verify this speculation, we designed and conducted some rescue experiments. First, the expression of *SH2B1* was increased using pcDNA3.1/*SH2B1* (Fig. 4A, *P* < 0.01). CCK‐8 assay delineated that the proliferation restrained by *BBOX1‐AS1* silencing was recovered with transfection of pcDNA3.1/*SH2B1* (Fig. 4B, *P* < 0.05, *P* < 0.01), which was further confirmed by colony formation assay (Fig. 4C, *P* < 0.01). Later, TUNEL assay and flow cytometry analysis revealed that *BBOX1‐AS1* knockdown‐induced enhancement on cell apoptosis was diminished by *SH2B1* overexpression (Fig. 4D,E, *P* < 0.01). According to wound healing assay, the up‐regulation of *SH2B1* offset the repression caused by *BBOX1‐AS1* depletion on cell migration (Fig. 4F, *P* < 0.01). In transwell assay, the invasion hampered by silenced *BBOX1‐AS1* was abrogated by *SH2B1* overexpression (Fig. 4G, *P* < 0.01). In brief, *BBOX1‐AS1* functioned as a ceRNA to promote CRC progression by targeting the *miR‐361‐3p*/*SH2B1* axis.

## Discussion

Previous studies have confirmed the pivotal status of lncRNAs in the process of tumor lesions [14, 15, 16]. Increasing lncRNAs are found to be involved in human tumor progression, including CRC [17, 18]. For example, the increased expression of GHET1 was tested in CRC samples, and CRC cell proliferation and invasion were inhibited on GHET1 knockdown [18]. In addition, down‐regulated PCAT1 induced cell apoptosis, arrested cell growth cycle, repressed proliferation, and hampered cyclins and c‐Myc expression in CRC [19]. In this study, the biological function and mechanism of *BBOX1‐AS1* in CRC were investigated, and results implied that *BBOX1‐AS1* displayed a considerably up‐regulated expression level in CRC cells. Moreover, with the transfection of sh‐*BBOX1‐AS1*, CRC cell proliferation, migration and invasion were dramatically restrained, whereas the apoptosis was observably promoted. These discoveries verified the oncogenic property of *BBOX1‐AS1* in CRC.

Based on the earlier findings, the mechanism mediated by lncRNA needs to be further researched. miRNA, with 22–24 nucleotides in length, was known as a critical regulator in biological processes through being sponged by lncRNA and targeting mRNA [20]. For instance, lncRNA XIST acted as an oncogene to boost CRC cell proliferation and reduce cell apoptosis through sponging miR‐132‐3p, and MAPK1 functioned as a target gene of miR‐132‐3p [21]. lncRNA MIAT, a sponge for miR‐29a‐3p, regulated the biological behaviors of gastric cancer cell by up‐regulating HDAC4 expression [22]. lncRNA CCAT1 activated cisplatin resistance via a mechanism relating to the miR‐130a‐3p/SOX4 axis in non‐small cell lung cancer [23]. With further exploration of the molecular mechanism in this study, we found that *BBOX1‐AS1* was mainly distributed in the cytoplasm of CRC cells and functioned as a sponge for *miR‐361‐3p*. Meanwhile, *miR‐361‐3p* expression was lowly expressed in CRC cells and negatively modified by *BBOX1‐AS1*. All of the data suggested that *BBOX1‐AS1* exerted the role of tumor facilitator by sponging *miR‐361‐3p*.

*SH2B1* was commonly recognized as an oncogene in multiple cancers. For example, *SH2B1* was identified as a risk factor in gastric cancer and stimulated its progression [24]. *SH2B1* enhanced the EMT process in lung adenocarcinoma through the IRS1/β‐catenin axis [25]. *SH2B1* promoted cell proliferation in non‐small cell lung cancer via phosphoinositide 3‐kinase/Akt/mechanistic target of rapamycin signaling cascade [26]. In this study, *SH2B1* was found to be highly expressed in CRC cell lines and targeted by *miR‐361‐3p*. Furthermore, *miR‐361‐3p* negatively regulated the expression of *SH2B1*.

In conclusion, this research revealed that *BBOX1‐AS1* promoted CRC progression by sponging *miR‐361‐3p* and up‐regulating *SH2B1*, which suggested a *BBOX1‐AS1*/*miR‐361‐3p*/*SH2B1* axis in CRC and provided a promising insight for CRC treatment.

## Conflict of interest

The authors declare no conflict of interest.

## Author contributions

J. Liu conceived and designed the project. JZ and ZX acquired the data. J. Luo and XW analyzed and interpreted the data. J. Liu wrote the paper. All authors approved final manuscript.
